# Acceptability of HIV self-sampling kits (TINY vial) among people of black African ethnicity in the UK: a qualitative study

**DOI:** 10.1186/s12889-018-5256-5

**Published:** 2018-04-13

**Authors:** C. Dodds, E. Mugweni, G. Phillips, C. Park, I. Young, F. Fakoya, S. Wayal, L. McDaid, M. Sachikonye, J. Chwaula, P. Flowers, F. Burns

**Affiliations:** 1 London School of Hygiene and Tropical Medicine, London, UK; 2 University of Glasgow, Glasgow, UK; 3 University College London, London, UK; 4 University of Edinburgh, Edinburgh, UK; 5 UK Community Advisory Board for HIV and iBase, London, UK; 6 BHA for Equality, Manchester, UK; 7 Glasgow Caledonian University, Glasgow, UK; 8 Royal Free London NHS Foundation Trust, London, UK

**Keywords:** HIV, Testing, Ethnicity, African, Self-sampling, Feasibility, United Kingdom, Qualitative

## Abstract

**Background:**

Increasing routine HIV testing among key populations is a public health imperative, so improving access to acceptable testing options for those in need is a priority. Despite increasing targeted distribution and uptake of HIV self-sampling kits (SSKs) among men who have sex with men in the UK, little is known about why targeted SSK interventions for black African users are not as wide-spread or well-used. This paper addresses this key gap, offering insight into why some groups may be less likely than others to adopt certain types of SSK interventions in particular contexts. These data were collected during the development phase of a larger study to explore the feasibility and acceptability of targeted distribution of SSKs to black African people.

**Methods:**

We undertook 6 focus groups with members of the public who self-identified as black African (*n* = 48), 6 groups with specialists providing HIV and social services to black African people (*n* = 53), and interviews with HIV specialist consultants and policy-makers (*n* = 9). Framework analysis was undertaken, using inductive and deductive analysis to develop and check themes.

**Results:**

We found three valuable components of targeted SSK interventions for this population: the use of settings and technologies that increase choice and autonomy; targeted offers of HIV testing that preserve privacy and do not exacerbate HIV stigma; and ensuring that the specific kit being used (in this case, the TINY vial) is perceived as simple and reliable.

**Conclusions:**

This unique and rigorous research offers insights into participants’ views on SSK interventions, offering key considerations when targeting this population.. Given the plethora of HIV testing options, our work demonstrates that those commissioning and delivering SSK interventions will need to clarify (for users and providers) how each kit type and intervention design adds value. Most significantly, these findings demonstrate that without a strong locus of control over their own circumstances and personal information, black African people are less likely to feel that they can pursue an HIV test that is safe and secure. Thus, where profound social inequalities persist, so will inequalities in HIV testing uptake – by any means.

**Electronic supplementary material:**

The online version of this article (10.1186/s12889-018-5256-5) contains supplementary material, which is available to authorized users.

## Background

In the UK heterosexuals of black African ethnicity^1^ are estimated to be between 20 and 40 times more likely to have HIV infection than the rest of the national population. Similarly, with disproportionate diagnoses reported among non-UK born homosexually active men, we would expect black African men who have sex with men (MSM) to be more at risk of HIV than their non-African counterparts [1]. In addition, black African people are overrepresented among those who present to UK HIV services with advanced disease accompanied by the myriad public health and personal wellbeing implications associated with delayed presentation [1].

Although a considerable proportion of HIV testing in the UK is undertaken through sexual health clinics, these settings tend not to be regularly attended by black African people [2, 3] and HIV screening in antenatal services is only accessed by pregnant women. Primary care services are utilised by black African people, however opportunities for earlier HIV diagnosis in such settings are often missed [4]. Historically there have proven to be many social issues that prevent timely HIV testing among black African people including concerns about: confidentiality, stigma and discrimination and fear of HIV diagnosis [5–9]. In addition, wider structural issues can keep black Africans distanced from HIV prevention, diagnostic and treatment services. These include, but are not limited to: poverty, under-employment and lack of childcare [5] which pose challenges for clinic accessibility; reticence among non-specialists to offer HIV testing [10, 11], accompanied by a lack of political will and a lack of African representation in the development of policy and services [8]. These are issues that have resonance across the global African diaspora (see for instance [12, 13]) meaning that this work also has the potential for international application. Given the increasing range of innovations in testing approaches and technologies, this work comes at a crucial time when policy makers and public health specialists require an up-to-date evidence base about new options and how they are likely to be perceived among black African people both in the UK and across the international African diaspora. By providing a clearer understanding of these perceptions, and the specific contexts in which they arise, it is possible to build an evidence base for service commissioning and policy that is premised on empirical evidence, rather than making the assumption that what works for one key population will be easily translated for another.

### Emergent HIV testing technologies

Emergent HIV testing technologies are often presumed to have the potential to improve take-up, by circumventing some of the barriers to testing outlined above (particularly privacy and stigma), yet technological change will have little impact for those who do not perceive the need to have an HIV test or are fearful of the results in the first place. Nonetheless, opportunities for improved access to testing are emerging in the expanded range of HIV testing options, accompanied by studies of some key population groups’ perspectives on the increasing availability of community-based point of care testing and self-sampling kits (SSKs) in the UK [14–16] and internationally [17–23]. There is considerable optimism that widened access to HIV testing may increasingly normalise the practice. Within this context, in April 2014 HIV self-testing (HIVST) kits became licensed for UK use, although to date their cost coupled with difficulties monitoring outcomes for users, means they have not yet been widely commissioned by the National Health Service (NHS). In the meantime, both oral and dried blood spot self-sampling for HIV have been shown to be acceptable among MSM [18, 24].^2^ There has been a growing expectation that SSKs will provide an acceptable and feasible alternative to clinic attendance, which may increase testing among MSM in the UK [25, 26] as well as among young men more broadly [27].

This paper reports findings from the development phase of a larger study which sought to establish whether embedding both the offer and provision of SSKs within existing services could also be a cost effective and acceptable means of increasing HIV testing uptake among all black African people, by increasing awareness about the benefits of testing, as well as improved knowledge of the range of testing choices on offer. A separate output arising from this study covers methodological rationales as well as findings across all project phases in greater detail [28]. Prior to this work being initiated, there was little evidence to support the acceptability or feasibility of targeting provision of SSKs among black Africans in the UK. One pilot study run by Terrence Higgins Trust/HIV Prevention England and Dean Street At-Home has documented success in reaching black African people through internet based SSK distribution [29, 30]. Though the study had greater success in uptake among MSM than black African people of all sexualities, one phase found that 9.8% of the 7761 SSK requested were by black Africans; 7.3% of which were returned, with a positivity rate of 2.6% within this specific sub-group. A systematic literature review undertaken to inform this wider study demonstrated that there was a limited body of empirical research undertaken on the use and perception of SSKs in general [28]. It also revealed a particular lack of focus upon members of migrant ethnic minorities which would enable an account to be given of the particular social contexts that influence their attitudes and experiences. To our knowledge, our study presents the first extensive qualitative data on the perceived appropriateness and feasibility of the offer, provision and use of SSKs as targeted community HIV prevention for migrant black African people within the context of existing services in their country of resettlement. It therefore has direct utility for those who plan and commission public health interventions meant to benefit this population; while also enabling greater reflection on how these technologies are likely to fit into overarching public health policy environments.

## Methods

The data described here are taken from the formative development phase of the larger project described and rationalised briefly above.^3^ Twelve focus group discussions (FGDs) and nine one-to-one interviews were undertaken between September and December 2014 in Glasgow and London with specialist providers of services to African people, and groups of black African people not working as specialist service providers (members of the public, referred to subsequently as *non-specialists*). These two cities reflect areas of divergent HIV prevalence, as well as different forms of health system infrastructure within the UK.

### Non-specialist FGDs (3 in London/3 in Glasgow)

Purposive sampling captured diversity of gender, age, region of origin and HIV testing experience. Participants were recruited via social media and African embassies in London, as well as among university student groups, and community based organisations in both cities. Participants were eligible if they self-identified as being black African and were aged 18 years and over. Eligibility screening was undertaken by telephone in order to initiate a relationship between researcher and participant, and to clarify the aims and process of data collection.

In order to ensure the facilitation of diverse and salient perspectives in these group settings, one of the FGDs was comprised only of people under the age of 30, another comprised African men only and a further group was comprised of people with diagnosed HIV. We did not purposively assemble an African MSM group as the intervention offers were not based on this parameter. Also targeted intervention research related to SSKs had already been undertaken in the UK to benefit MSM of all ethnicities [26]. In total, 48 self-identifying black Africans participated, representing a relatively heterogeneous sample of individuals comprised of 28 men and 20 women; with ages ranging from 18 to 60. Participants hailed from various regions, including: East Africa (*n* = 17), Southern Africa (*n* = 10), West Africa (*n* = 10), Central and North Africa (*n* = 3) with some born in the UK, Europe or USA (*n* = 7, missing = 1)). Our sampling strategy also ensured that we were able to include the views of participants who had never tested for HIV (*n* = 19). Although we did not screen for sexuality amongst participants, a minority (approximately 4) made it clear during the focus group discussion that they were men who have sex with men. The nature of our varied recruitment strategy means we are unable to estimate a refusal rate.

### Specialist service provider FGDs and interviews (3 in London/3 in Glasgow)

Purposive sampling was again used to ensure service providers were from a range of professional backgrounds. Primary care providers were recruited via the Primary Care Clinical Research Networks in London and through established working relationships with members of the research team. Community workers in both cities were recruited from organisations with extensive experience of delivering HIV prevention and care as well as a range of other non-HIV specific services to black Africans. The research team approached pharmacies within areas with high concentrations of African residents in both cities, with support from local pharmacy associations. Almost all specialist FGDs comprised those from diverse working backgrounds in order to elicit contrasts within working and experiential contexts. One to one interviews with HIV clinicians, HIV service managers and commissioners were also conducted.

In total, 53 service providers participated, including: General Practitioners (GPs) (*n* = 8), Pharmacists and Pharmacy Assistants (*n* = 10), Nurses (*n* = 3), Health Care assistants (*n* = 1), HIV community based organisation staff (*n* = 15), other African service providers (*n* = 5), African faith leaders (*n* = 3), HIV consultants (*n* = 5), as well as service funders, policy makers and commissioners (*n* = 3). There was a fairly even gender split amongst specialist participants (29 men and 24 women). Using these varied recruitment methods, it is difficult to report an accurate refusal rate, although the following refusals were recorded from amongst our targeted invitations (community organisations *n* = 3, general practitioners *n* = 3, pharmacists *n* = 22); and cancellations among those registered to attend were rare (1 pharmacist and 1 nurse).

Semi-structured topic guides and planned activities (available at: http://www.haus.org.uk/about/about-haus) prompted discussion about: accessibility and perspectives toward HIV testing in general; perspectives on SSKs and their utility within this population; practicalities and acceptability of targeted distribution in a range of community settings used by black African people; and potential procedures for sample return and clinical governance. The researchers involved in data collection (CD, EM, SW, CP, GP and IY) sought to create a balance between the a priori issues outlined above while also harnessing participant-led articulation of perspectives, social norms and discourses [31].

**Fig. 1:**
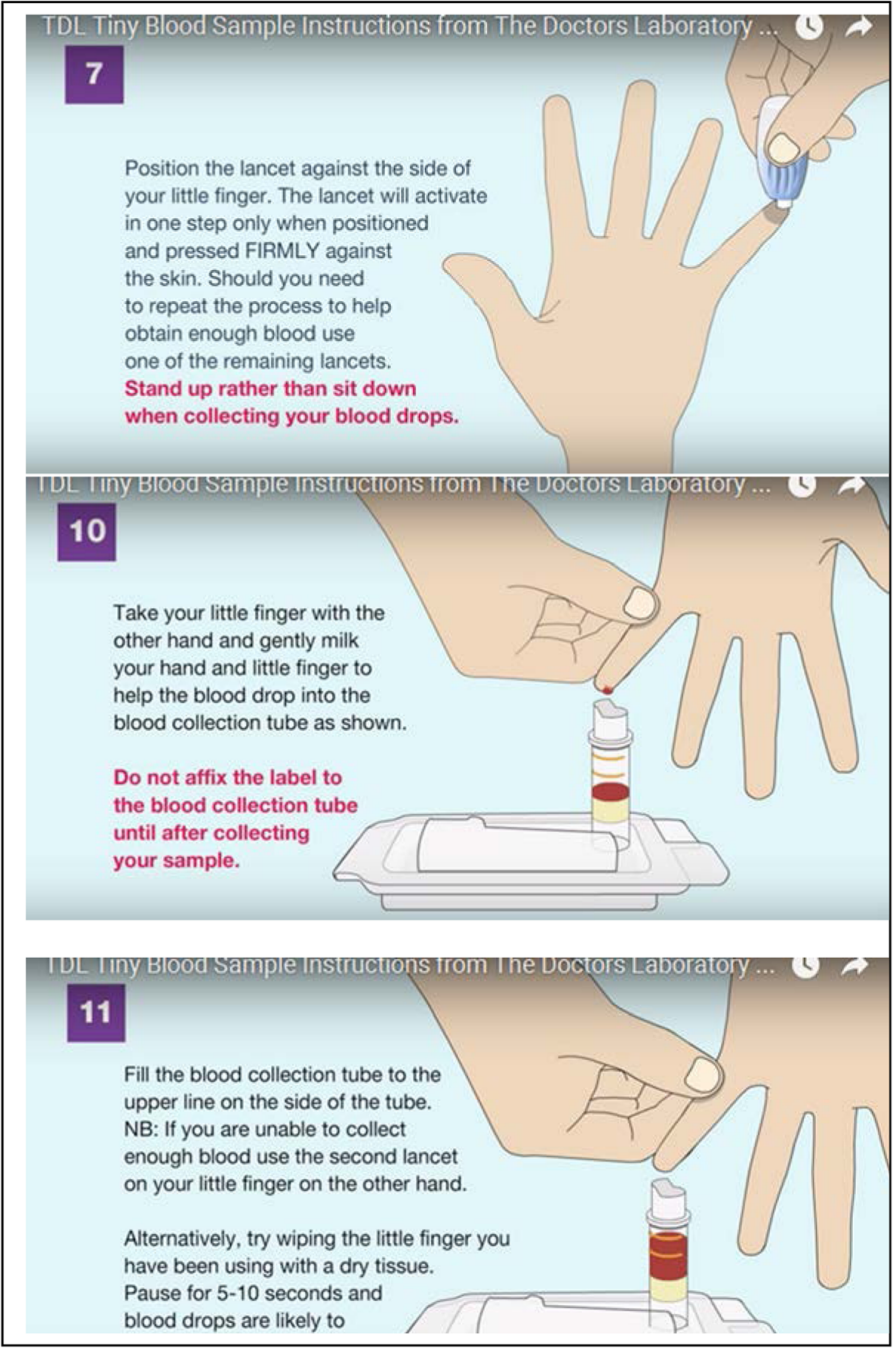
Stills from corporate instruction video

In all focus groups, participants were shown a video (See Fig. 1) produced by the producers of the TINY Vial SSK (http://www.tdlpathology.com/test-information/test-service-updates/tdl-tinies) Many were also shown an instructional video developed by a community organisation (https://www.youtube.com/watch?v=FSm0zP1TGUo) on self-use of dried blood spot sampling kits, for contrast between available self-sampling options. We displayed, distributed and discussed TINY Vial kits in all groups. As use of oral fluid kits would not be possible within the governance structure of our study,^4^ no oral based kit was demonstrated during the groups, however, potential benefits of their use were raised by participants.

Each focus group was run by two expert qualitative researchers taking it in turns to lead the discussion and observe/take field notes [32].^5^ These took place either on the premises of participating research institutions, or in community based organisations with only the two researchers and participants present.

In addition, we undertook nine one-to-one interviews with HIV clinicians, HIV service commissioners and service managers at their places of work (*n* = 4 London and *n* = 5 Glasgow) in order to ensure data saturation and also to avoid their presence in focus group discussions unintentionally suppressing colleagues’ input.

Both the public non-specialist participants and the specialist service providers attending focus groups were offered reimbursement for their travel and their time given to the study. Each interview lasted between 30 and 45 min while the FGDs lasted between one and a half to 2 h. Digital audio recordings of interviews and FGDs were transcribed verbatim, and field notes were taken in order to supplement and support the transcripts.

### Data analysis and conceptual framework

This work is rooted in an understanding that simply undertaking interventions to increase knowledge of HIV and its modes of transmission are insufficient to ensure uptake of testing among the black African population in the UK [33]. Alongside information, people need the motivation as well as the opportunity and capacity to act in ways that reduce the risk of HIV transmission and acquisition (including determining one’s HIV status) [34]. In the specific context of timely HIV testing, this means that people need to feel that having a test is worthwhile, beneficial and straightforward, and that the act of having a test in and of itself should not carry negative repercussions. In structural terms this will require an assured and confident HIV testing infrastructure in which professional service providers possess the capacity and confidence to offer HIV tests to those who are likely to be most at risk of acquisitions, including black African people. Practical elements arising from the COM-B model of behaviour change [35] further supported our data analysis and planning for subsequent intervention development phases. It was furthermore essential to acknowledge across the course of project design, data collection and analysis, that: a) ‘black African people in the UK’ do not represent a singular homogenous group, so diverse approaches to establishing acceptability (as well as approaches to increasing HIV testing access) were likely to be required; and b) that the accessibility, uptake and architecture of HIV testing among black African people in the UK could only be considered within its wider social, political and cultural context [8, 36].

Thus, we sought to describe the range of diverse views and broad commonalities within participants’ discussions and interviews - focusing on the ways these relate to social mores, norms and key cultural concerns. Where considerable differences between groups were obvious these are noted and are made explicit.

We used NVivo 10 for overall data management, and initial thematic analysis was undertaken collaboratively by seven members of the team (CD, EM, LMcD, PF, GP, IY, FB) using a Framework approach [37]. We applied both inductive and deductive approaches, from which initial themes emerged, including: the feasibility and accessibility of HIV testing, existing knowledge and uptake of SSKs, the practicalities of distribution as well as cross-cutting themes which influenced all of these, particularly those concerned with trust and HIV-related stigma. These were then refined to devise a more detailed participant-led, inductive, thematic framework used for coding by CD and EM (see Additional file 1 for a detailed coding tree). Researcher-team discussions and iterative analysis focussed upon the internal coherence and face validity of the resulting analytic structure. Anonymised quotations are used to illustrate the analysis.

## Results

The results are divided into the three key elements of an integrated intervention to offer SSKs in community settings, that is: issues arising in targeting SSK offers to Africans; expanding the range of testing options on offer; provision of SSKs in ways that manage privacy and HIV stigma concerns; and the practicalities of using SSKs.

### Offering SSKs to Africans

While there was enthusiasm around the abstract notion of distributing SSKs to disproportionately benefit black Africans, it is worth noting there may have been some degree of selection bias within the sample, given that people were told about the topic of research at the point of research recruitment.

The SSK was often described by participants as an important means of bolstering the array of HIV testing options on offer for black African people.

*I like the idea of home sampling because it gives more choice, flexibility and opportunity for people to have an HIV test. So for example, if people are worried about confidentiality, they can do the test in the privacy of their own home. And even though they still have to send the result in to a lab, at least it’s not done through a third person, having to disclose their history and why they’re worried about HIV.* [specialist interview]

Although the kits were certainly not regarded as a panacea that would be able to resolve all social and practical barriers to testing in this population, specialists and non-specialists alike were interested in the extent to which the SSK offered an opportunity for increasing individuals’ decision-making about the setting and context in which they would take a test, perhaps contributing to improved health autonomy overall. A small number (of mainly service providers) hoped that widespread availability of such kits within black African communities might help to relieve HIV stigma at a deeper, structural level, as they could help to ‘normalise’ HIV testing. Not everyone agreed, as it was also mentioned that increasing the extent of privacy through the use of SSKs may serve to hide HIV even further away.

A key issue that emerged in relation to making the offer of SSKs to black Africans was the concern such behaviour would be perceived to be driven by racism and discrimination, a matter raised by both specialist and non-specialist participants as being a reflection of the problematic power relations in these settings. In addition to the divisiveness that such an approach might be seen to represent, there was also a concern that service providers undertaking ethnic targeting might do so simply based on skin colour, and that such an approach would be complicit with the homogenisation of highly diverse cultures and communities. Additionally, some participants felt that such judgements could be based on mis-guided understandings of whether someone looks ‘black enough’ to be considered as African. All of these contributions demonstrated participants’ recognition of, and concern about, the way that power imbalance pervades African people’s engagement with HIV testing (a behaviour that is still considerably stigmatised in African communities) and with health services in general. Many African participants (both specialists and non-specialists) described previous experiences of racism, being patronised and being concerned about a lack of fully informed consent in health settings, and this extended to a concern about providers’ judgement and communication strategies when it came to HIV. Having said this, there were also participants who pointed out that targeting those groups that the epidemiological data shows to be in greatest need, is the best use of limited resource:

*Participant 1: I have trouble just targeting just Black communities with that kit, for me it has to be universal for everyone I wouldn’t like to just target a specific population group.*

*Participant 2: This is screening! If the epidemiological studies that there’re high prevalence and new incidence rate in that race, in that particular community, that is really…really where resources should be.* [service provider group]

Some suggestions to help ameliorate potential harm from targeting included: ensuring that African people are involved in designing and delivering targeted interventions; ‘bundling’ HIV testing in with other ethnically targeted interventions such as sickle cell and blood pressure screening; and ensuring that publicly available materials do not flag up the link between Africans and HIV prevalence, as this information can be misconstrued and misused by a wider audience.

Quite apart from the matter of targeting SSKs based on African ethnicity, were a series of further concerns about the fact that SSKs may not guarantee African users the sort of sustained interpersonal engagement and support traditionally tied to pre- and post-test HIV counselling. So although this technology had the potential to offer greater autonomy, flexibility and choice in HIV testing, worries about the risk of suicidal thoughts and a potential for self-harm by those who were either waiting for their results, or being told them by telephone, emerged in a considerable proportion of groups and interviews. In particular, participants expressed concern about cases where a user may be informed by telephone of a reactive result, requiring confirmatory testing in the following days. Those expressing these safeguarding concerns felt that being in the physical presence of a professional for discussion of test results could help to reduce anxiety, lessen the change of immediate harm, and assure linkage to support and care services for those with less capacity to seek these out on their own; and that for these reasons, SSKs represented a less ‘safe’ option for users.

*I mean generally I’m kind of definitely, sort of, pro this sort of testing but I think that’s something that has to be thought about that anyone can pick this up and they might not be in a state of mind where they’re okay to receive that news.* [specialist group]

Comments such as this reveal an underlying concern that SSKs increase autonomy at the expense of clinical systems’ control, as these are frequently regarded as the only means of ensuring extended contact with prevention, treatment and support services. An assumption is therefore made that no matter what an individual tester’s preference for a convenient service, that face to face services (preferably accompanied with a point of care test with rapid result) should be deemed to be the superior option. As such, in all focus group discussions and several interviews there was a clear preference expressed for the offer and discussion of HIV testing to always involve direct contact with a highly skilled specialist provider, with the capacity and resource to undertake immediate risk assessment followed up by pre-test counselling and a point of care test (where appropriate), so that an immediate result could be delivered within the same consultation event.

*I think it's very clinical [referring to the SSK], I personally would probably rather go to my GP and say here I am. And also there's something very human about having somebody…I remember my first HIV test, I was petrified, I had written my will, I couldn't think about anything else. I was stressed so how on earth I could have done this with ten drops of bloods and then post and then label and then date of birth and then this without help. What helped that day, there was a nurse who was able to talk to me. And I didn't feel alone. This [the SSK] was a very lonely procedure.* [specialist group]

Some did recognise that following on from a risk assessment discussion, a specialist service provider might come to the realisation that an SSK is the most appropriate option for a particular individual. However, most held that it was unlikely to be the most appropriate first-line HIV testing intervention for most black African people. What most participants made clear is that the ideal HIV testing intervention is one that assures the availability of face to face support before, during and after any HIV test that takes place. The SSK was regarded as a technology that potentially would make users feel detached from support. These views are of particular interest given current guidance suggesting a reduced need for pre-test counselling when HIV testing [38]. To this extent distribution of SSKs was frequently characterised as being a ‘poor relation’ to point of care HIV testing.

*If you're going to start offering it as an anonymous kind of thing that's not connected to services, well, what's the reason for doing that? What problem are you trying to solve by offering that?* [specialist interview]

Right from the outset, then, these findings highlight a key set of tensions that emerge with screening technologies which prioritise freedom and autonomy. While these features are regarded as having potential benefits in terms of accessibility, what these technologies are seen to lack is an assurance of immediate access to support interventions. In addition, participants expressed concern about the delay in the communication of results (and the attendant stress that could result), as well as procedures for communication of results that might lack clinical robustness. To this extent, many felt that in settings where a trained specialist was already afforded the space and privacy to introduce HIV testing as an option, priority should be given to the offer of a point of care HIV test in such a setting, rather than an SSK.

### Provision of SSKs: the primacy of privacy and stigma avoidance

All participants were clear about the profound challenges that HIV stigma presents in terms of willingness to engage in HIV prevention and testing interventions, in the UK just as much as anywhere else. The implications of this can be even more profound for those who are marginalised by uncertain immigration status, precarious or under-employment, living in a society pervaded by racism and xenophobia [7]. HIV-related stigma at structural and interpersonal levels represents a clear challenge to successful intervention to promote HIV testing within this sub-population. It predicates against self-perception of risk, and even where an individual may overcome this, it then promotes fear about being seen to access an HIV testing service, because of the social implications that may follow.

*They rather, you know Africa, we rather die than to expose ourselves. You understand, we rather… If not for my pregnancy as [XXX] said, I wouldn't go, I wouldn't. Do you understand?* [non-specialist group]

As a result, our analysis revealed a profound (and often conflicting) set of accounts about the need to afford privacy in order to discuss and facilitate the undertaking of HIV testing among black African people. These are by no means new concepts for the field, but this emergent technology raises a number of new considerations, particularly in the way that the demand for privacy emerges from and potentially impacts upon HIV-related stigma. For instance, non-specialist participants were highly sceptical about offers and distribution of SSKs by community pharmacists and primary health care practitioners that would be free of judgment or microagression [39], alongside concerns that confidentiality might be breached (in this way, the power dimension emerges again).

As a result, the privacy and confidentiality afforded to individuals in each potential SSK distribution setting was the overriding factor that helped participants gauge its suitability. To make matters more complex, for every potential SSK distribution setting that was proposed as having ideal privacy protections by some participants, others were quick to disagree, by examining a new set of confidentiality concerns. Therefore, while some saw GPs as having an ideal combination of privacy and medical expertise, others worried that Home Office officials could be notified of an outcome, and there were concerns that even the discussion of an HIV test could persist on a medical file with negative consequences.

*But for those who have come through that route, there were also a whole package of concerns about if you disclose your name and health status, would that affect the asylum decision. [specialist interview]*

While some argued that ordering such a kit to be delivered through the post at home might be ideal, this suggestion was almost always vetoed by others who felt that most black African people in the UK did not live alone, and the arrival of such a kit in the post (or even the carrying of a package that is distributed in the community) would always elicit questions about what is inside.

*The kids can…you know kids, they like playing and it’s, like…I don’t know, maybe neighbour kids, the one who reads, is there and stuff. And then he starts, hey mum, I was next door, I’ve seen this.* [non-specialist group]

These data demonstrate that we cannot underestimate the extent to which considerations of privacy, and its limits, are at the centre of considerations for SSK feasibility and acceptability among black African people in the UK. Our participants were at pains to emphasise that many in this target population will lack the power to act as independently as such testing models often presume, as demonstrated in the comment below.

*Another thing that’s comes from, I think, this would make, raise conflict between, for example, a man, wife and husband, for example they are, let’s say, an example of, there are those, kind of, communities, if a man doesn’t want something, that mean how, a woman can’t do anything, so, a woman can want to, to just do the test, but there’s no way, nowhere to hide […] what about sending it and receiving the, the, the result? […] I think if the man is okay with what you are doing, everything in the household is fine, it’s only when the man is against what, you know, is being, you know, the information that is being given out at that time, maybe they say home testing kits, you are watching telly and automatically the man says, oh, the man says, oh, this is not going to happen in my house, and with you as a woman, you want to do it, that’s a no-no in your house, so, it’s really hard for them.* [specialist group]

The prevalence of such issues present considerable challenges to the introduction of a technology that is meant to assist in circumventing the problems of low HIV testing uptake among black Africans. They remind implementers to be cautious about not introducing new problems while trying to address existing ones.

At the same time, among the beneficial elements that participants associated with SSKs, privacy, discretion and the capacity to determine one’s own status in an environment of relative anonymity were regarded as considerable strengths, particularly among those taking part in non-specialist focus groups. Being able to use a kit privately was regarded a means of enhancing willingness to test among those who were unlikely to: know about or use a sexual health clinic, raise HIV in a clinical setting, or seek out community based testing – which all relate to the potential for increased autonomy already discussed above. These mechanisms for achieving increased privacy through new routes of access to HIV testing were therefore recognised as ways to help users avoid, or at least better manage, the widely-perceived stigma which can discourage many from seeking out an HIV test in the first place.

*[It’s] quite hard for some people to go and approach GPs or doctors to explain their situation. Like myself, I've been thinking about it. It's been in my mind for a long time to do a test, because I've been hearing people, I've been watching this, I've been… you know what I mean, media’s talking about it, so I don’t even know my status, but when something like this came up, if it's, like you said, I think it's an opportunity for people like me to take the chance to do it.* [non-specialist group]

Interventions that increase knowledge of SSKs (by introducing them in embedded services) and demonstrate their convenience (particularly if introduced sensitively by skilled service providers) were therefore hailed by some as increasing the likelihood of HIV testing among Africans.

*… especially with location, where people live, they don’t have that time to go to the hospital and go through the whole process of getting a HIV test.* [non-specialist group]

At the same time, there was also some concern expressed that this extended privacy meant SSKs might contribute unwittingly to ‘keeping HIV underground’, providing cover to those who desired secretive means of confirming whether or not they are infected.

### Practicalities of using HIV SSKs

Most non-specialist participants were unaware of SSKs prior to taking part in this research, and only a handful disclosed having used one in the past – so existing levels of knowledge were low. Some had heard of instant result self-testing, as there were national media reports about that technology being licenced just before the period of research; and a sub-set of these were then surprised to learn of the requirement to take a sample, post a vial of blood and await laboratory results. Participants were surprised at the volume of blood (400 μl) required for a sufficient TINY Vial sample. In particular, many specialist providers stressed that they did not think that most black African members of the public would be able to produce enough volume for a viable sample – so there was concern about the way the demands of this kit might influence willingness to use it.

*I mean, we do health checks and we take blood from the finger and our machines just been changed to take a much smaller sample, we have to take 40 microns of the blood, not a big amount which is why I kind of, I was a bit shocked at this. And just getting that amount of blood is actually sometimes quite traumatic for a person.* [specialist group]

In addition to discussion about fear of needles and blood inhibiting self-sampling, a few pharmacists, nurses and GPs held the view that physiologically, their black patients may have difficulty producing fingerprick samples because of a perception that they would have thickened skin on the fingertips.

Many participants across all focus groups felt that for these reasons, as well as complexities of using, labelling and sending the sample in a vial, the requirements of the TINY kits were too onerous for most people to use correctly – and some might just give up on it before they even begin, due to perceived complexity.

*I think that will be quite tricky. Certainly, I don't think it’s one that you can tell them it’s that easy to do […] maybe if the test was simpler.* [specialist group]

*Can I be very honest? I don’t like this, and the reasons why I don’t like it is because it isn’t simple… It isn’t easy and, of course, this will not be popular. I think this is an attempt to make people laboratory people. By the time people put a jab and then put their hands and blood starts dripping, one, two, three, four, five, six, seven, eight, up to 20, I find it a bit… very, very cumbersome. It makes it very, very… It’s liable to a lot of mistakes. And so what do you then do?* [non-specialist group]

Furthermore, a number of participants expressed concern that SSKs could be easily contaminated by users, perhaps by touching the top of the vial or the lid, or not cleaning the puncture site sufficiently. Others questioned the robustness of the technology and procedures on offer to the public, and felt users may worry about samples being mismatched at the laboratory. So there were issues with a lack of trust over direct engagement with providers of laboratory services. A few specialists and non-specialists also conveyed an (erroneous) concern that unskilled users might cause onward transmission through a blood spill.

In many of the discussions with specialists, they raised comparisons between these HIV SSKs and a range of kits for other conditions that are now designed for self-sampling. These comparisons highlighted that a range of kits intended for self-sampling had not been a great success (chlamydia and bowel screening kits in particular were perceived as under-used and not cost effective). In contrast, HIV specialist providers with experience using dried blood spot kits for HIV self-sampling among MSM were encouraged by the benefits that self-sampling could bring to African users.

## Discussion

These findings reveal that despite the clear sense that SSKs may increase access to HIV testing among African people in the UK, that both users and providers were cautious about the value they would really add to the current testing landscape, given their concern that SSK provision involves a low level of interaction with those who can provide specialist advice and support throughout the testing process. Such perspectives are connected with the view that all people accessing HIV testing (and in particular, black Africans in this instance) need to have a strong locus of control over their own information alongside the practicalities of kit use – and that many SSK processes would need strengthening in order to secure confidence and acceptability within this population. Also this study again raises a number of concerns from both providers and potential users, that the offer of SSKs in way that is intended to disproportionately benefit African people in community settings needs to be undertaken with considerable sensitivity to the HIV stigma that such targeting may exacerbate. This discussion section considers these issues in light of health behaviour change models as a means of considering how best to design targeted SSK interventions that are acceptable and feasible among black Africans.

In almost all current models of health behaviour change and models for influencing health it is recognised that the social ecology plays a significant part in enabling the desired behaviour to take place. The model that underpins this paper holds that people require *knowledge*, *will* and *power* to ensure their HIV prevention and testing needs are best met – and these have each been flagged throughout the presentation of the findings above [34] (also see [40, 41]). In this final section, we will consider how, in the case of SSKs, these three factors are likely to combine to influence decision-making and support action. Where there is a deficit in one area, the other two are necessarily diminished, and conversely, there any one of these factors is increased it improves the likelihood that the other factors will also be improved.

In the context of this work, *knowledge* relates to: awareness of a range of HIV testing options, information that enables a person to accurately assess their own HIV risk over the course of a lifetime, and knowledge of HIV transmission basics. This research project was not designed to assess the degree of such forms of knowledge at a community level, and there are other data sources that provide insights into this set of knowledge among this population [42]. In terms of the specific knowledge needs related to SSKs, despite their availability in the UK for a number of years, most specialists and non-specialists alike had no prior awareness of their availability or use. If there is a desire to increase uptake of this option among black African people, there is an acute need to improve information about availability for this population and those who provide them with services. To this extent the way in which the kits are offered will be key to helping to improve the knowledge base about their existence and utility.

When referring to *will* in this conceptual framework, we are referring to the perceived benefits and costs of a range of behaviour options and their alternates. Within this process of weighing up benefits and costs, each individual’s tipping point (within each instance of a decision being made anew) will vary depending on how much importance they place on the potential benefits, as opposed to how much significance they accord the potential harms. In many instances, this process is profoundly influenced by what we think is acceptable and important to those who are significant to us, by what we think our significant others would do in the same situation, and by how much we want to conform with our significant others [41]. Below we discuss a range of the findings described above in light of the way that they could influence willingness to use an SSK when offered it.

The issue of HIV-related stigma, and whether people had the skill, capacity and will to tackle it was perhaps the most influential determinant of the way they regarded the feasibility of uptake of these kits among black Africans. For some, the privacy and autonomy afforded by such kits made it a highly acceptable and worthwhile option, while for others the social risks associated with even being known to accept or carry such a kit were seen as an ongoing barrier to uptake. Concerns about what significant others (and even strangers) might think about a person who needed an HIV SSK were at the centre of most discussions.

At an infrastructure level, data from specialist participants also made it clear that there remains an ongoing tension between the desire to provide an increased range of HIV testing options to people with the greatest likelihood of undiagnosed HIV, while at the same time a concern that the further away from clinics that such technologies venture, the less ‘systems control’ that can be exercised over the outcome. These tensions between increasing autonomy at the cost of ensuring a singular and reliable pathway into HIV care are likely to have considerable impact on the willingness of specialist providers to make SSKs available to those most in need. Furthermore, health providers who do not specialise in HIV expressed particular concern about being perceived as racist if they target their African patients for HIV testing, and so an unwillingness to target SSK distribution to some subgroups in greatest need (for fear of the harmful repercussions) is clearly a further issue to be addressed. A final point to make about willingness to use the SSKs relates to the direct concern that the TINY vials would be too difficult to use, and that the volume of blood required would be off-putting to many people.

Finally, in order to act on any intention to undertake a health-related behaviour, people need to have the necessary material resources, skills and opportunities which offer them the capacity to make a free choice. Without this *power*, knowledge and will - on their own - are insufficient. The findings described above demonstrate the myriad of ways in which HIV-related stigma impacts on people’s capacity to act, particularly when they lack control in other areas of their life. Whether it is inequality within sexual relationships, or concerns about deportation that may cause people to fear the act of finding out their HIV status (or what might happen if the result was positive), there were many times that participants pointed to the range of social vulnerabilities experienced by black African people which prevents them from confronting their own HIV risk. There were also those who were strongly of the opinion that at least for some people, SSKs represented a possibility for liberation from these norms, because it shifted the locus of control over privacy, timing and results management towards the individual. Of course, such an approach was not regarded as being risk-free, but on balance, some participants regarded the benefits as greatly outweighing the potential harms. Yet others felt that this gain could be outweighed by those cases where vulnerable individuals might feel trapped, alone and powerless while waiting to hear of their result, and that telephone support in such a context was insufficient.

It is clear that in order to gain a reasonable foothold amongst the array of HIV testing options currently on offer, SSK interventions will need to better demonstrate how they add value (which could potentially include greater focus on distribution in settings where distributor expertise and/or privacy are minimal). In addition, those planning such interventions will need to take serious account of the implications of these findings as they seek to improve the knowledge, will and power of black African people in the UK who need to establish their HIV status.

### Limitations

These qualitative findings do not make a claim to broad generalisability, however, they were sufficient to enable the study team to take account of key issues in developing the protocol for the second phase of the larger study. Additionally, given the governance constraints of the research environment, we were not able to explore other SSK options (such as dried blood-spot kits, or those using oral saliva samples) in much detail within this study. This is because the TINY vial is the only SSK device and assay that is CE approved in the UK. We were unable to locate a service provider willing to accept the liability of proceeding with using a product off-license.

This means that participants’ views on SSKs were heavily influenced by their impressions of the TINY vial kit itself, and some of these issues could have been ameliorated or altered if they had been asked to consider other self-sampling technologies as comparators, for instance. Nonetheless, this paper has focused on a range of generic issues related to SSKs that would apply no matter what device or technology was used, including discussions about privacy, communication of results and HIV-related stigma. It is also worth noting that all participants in this study were drawn from urban environments, and it is possible that those in more rural or semi-rural locations might have different perspectives to bear on the utility of SSKs.

## Conclusion

These findings reveal that despite some general initial enthusiasm, SSK use and targeted distribution among black African service users and their providers in the UK is likely to be greeted with considerable ambivalence. Having undertaken the first in-depth study to explore the feasibility and acceptability of targeted distribution of these kits to black African people accessing existing services, we have found that although an improvement in the variety of HIV testing options is welcomed, this technology will not be a panacea for the range of issues that prevent timely testing, nor will it usually be preferable to face to face rapid testing with immediate opportunity for referral. We have collated an extensive range of specialist and non-specialist perspectives, subjecting these to rigorous thematic analysis in order to better understand how SSKs can be put to best use for this community. Policy makers, public health specialists and commissioners of HIV testing services require these insights to guide their decision-making on HIV testing options in the future. Furthermore, we expect those examining potential HIV testing technologies for use among the African diaspora in other high-income countries may find resonance with this work. Clinical practitioners and community organisations will also find that the themes of privacy, dignity and locus of control are worth exploring further before instigating their own targeted services for migrant groups.

## Additional files

Additional file 1: Thematic coding hierarchy. (PDF 426 kb)

## Abbreviations

FGDs: Focus group discussions
GPs: General practitioners
HIV: Human immune deficiency virus
MSM: Men who have sex with men
NHS: National Health Service
SSK: HIV self-sampling kit
UK: United Kingdom

## Footnotes

1 The term ‘Black African’ appears as an ethnic category in the UK Census, and is widely used in UK epidemiological contexts to denote those who are part of the sub-Saharan African diaspora. It can be used to refer to people who have themselves migrated from this region, or whose predecessors have done so. This is also a highly contested and imprecise term, as it relates at one and the same time to self-identification as well as external-identification of racial/regional categories which are far from straightforward, and which also intersect with nationalities as well as linguistic and cultural groupings.

2 HIV SSKs are designed for users to take their own sample and then post it to the laboratory themselves. Results are then usually communicated to the user via either SMS text (where results are negative) or by a phone call (where there was an insufficient sample or a reactive result). In UK settings, clinical governance over laboratory procedures, and results communication is overseen by those working in expert clinical settings, and where a sample is reactive, sufficient information is given to the user to encourage them to attend their nearest clinic as soon as possible for confirmatory venous sample testing. While HIV SSKs can use either blood or oral fluids, only the blood based TINY vial and associated laboratory assay is CE Marked for approved use in the UK; also no oral SSK is currently able to provide 4th generation testing as recommended by the British HIV Association guidelines.

3 More detail is available at: http://haus.org.uk/about/about-haus; with a full trial protocol available at: https://njl-admin.nihr.ac.uk/document/download/2007197

4 Given that there is currently no CE marked saliva-based HIV assay available in the UK, the study team was not permitted this type of ‘off label’ use.

5 CD, EM, SW, CP, GP and IY (all female Research Fellows / Lecturers with PhDs in medical sociology, social epidemiology or allied disciplines) conducted the data collection. All have considerable experience and training in the collection, management and analysis of qualitative data. Most members of the team also had considerable experience in the HIV field.

## References

[1] Kirwan P, Chau C, Brown AE, Gill ON, Delpech VC and contributors. HIV in the UK - 2016 report. London: Public Health England; 2016.

[2] Skingsley A, Yin Z, Kirwan P, Croxford S, Chau C, Conti S, Presanis A, Nardone A, Were J, Ogaz D (2015). HIV in the UK–situation report 2015: data to end.

[3] Mercer CH (2017). Personal communication.

[4] Burns FMJA, Nazroo J, Ainsworth J, Anderson J, Fakoya A, Fakoya I, Hughes A, Jungmann E, Sadiq ST, Sullivan AK, Fenton KA, SONHIA Collaboration Group (2008). Missed opportunities for earlier HIV diagnosis within primary and secondary healthcare settings in the UK. AIDS (London, England).

[5] Burns F, Imrie J, Nazroo J, Johnson A, Fenton K (2007). Why the(y) wait? Key informant understandings of factors contributing to late presentation and poor utilization of HIV health and social care services by African migrants in Britain. AIDS Care.

[6] Prost A, Elford J, Imrie J, Petticrew M, Hart G (2008). Social, behavioural, and intervention research among people of Sub-Saharan African origin living with HIV in the UK and Europe: literature review and recommendations for intervention. AIDS Behav.

[7] Dodds C (2006). HIV-related stigma in England: experiences of gay men and heterosexual African migrants living with HIV. J Community Appl Soc Psychol.

[8] Fakoya I, Reynolds R, Caswell G, Shiripinda I (2008). Barriers to HIV testing for migrant black Africans in Western Europe. HIV Med.

[9] Flowers P, Davis M, Hart G, Rosengarten M, Frankis J, Imrie J (2006). Diagnosis, stigma and identity amongst HIV positive black Africans living in the UK. Psychol Health.

[10] Davies C, Gompels M, May M (2015). Public and healthcare practitioner attitudes towards HIV testing: review of evidence from the UK. Int STD Res Rev.

[11] Deblonde J, De Koker P, Hamers FF, Fontaine J, Luchters S, Temmerman M (2010). Barriers to HIV testing in Europe: a systematic review. Eur J Pub Health.

[12] Blanas DN, Nichols K, Mulusew B, Lugg A, Kerani R, Horowitz C (2013). HIV/AIDS among African-born residents in the United States. J Immigr Minor Health.

[13] Gardezi FCL, Husbands W, Tharao W, Lawson E, Myers T, Pancham A, George C, Remis R, Willms D, McGee F, Adebajo S (2008). Experiences of and responses to HIV among African and Caribbean communities in Toronto, Canada. AIDS Care.

[14] Westrop SJ, James C, Edwardes D, Brady M, Gillespie A, Gill ON, Nardone A (2014). Testing history and risk behaviour of individuals requesting an HIV test through an online self-sampling service. AIDS 2014-20th international AIDS conference: July 20th -25th 2014; Melbourne, Australia.

[15] NICE (2011). HIV testing: increasing uptake in black Africans.

[16] Weatherburn P, Hickson F, Reid D, Hammond G (2006). Evaluation of the Department of Health funded fasTest HIV testing in the community pilot.

[17] Skolnik HPK, Binson D, Dilley J (2001). Deciding where and how to be tested for HIV: what matters most?. J Acquir Immune Defic Syndr.

[18] Spielberg F, Critchlow C, Vittinghoff E, Coletti AS, Sheppard H, Mayer KH, Metzgerg D, Judson FN, Buchbinder S, Chesney M (2000). Home collection for frequent HIV testing: acceptability of oral fluids, dried blood spots and telephone results. HIV Early Detection Study Group. AIDS (London, England).

[19] Spielberg FCC, Vittinghoff E, Gross M, Doherty-Iddings P, Scotti R (2001). Slow diffusion of home HIV-specimen collection. Sex Transm Dis.

[20] Colfax GLJ, Bindman A, Vittinghoff E, Vranizan K, Fleming P (2002). What happened to home HIV test collection kits? Intent to use kits, actual use, and barriers to use among persons at risk for HIV infection. AIDS Care.

[21] Greensides DBR, Lansky A, Sullivan P (2003). Alternative HIV testing methods among populations at high risk for HIV infection. Public Health Rep.

[22] Sharma ASP, Khosropour B (2011). Willingness to take a free anonymous home HIV test and associated factors among internet-using men who have sex with men. J Int Assoc Phys AIDS Care.

[23] Sharma ASR, White D, Sullivan P (2014). Acceptability and intended usage preferences for six HIV testing options among internet-using men who have sex with men. Springerplus.

[24] Dodds JP, Johnson AM, Parry JV, Mercey DE (2007). A tale of three cities: persisting high HIV prevalence, risk behaviour and undiagnosed infection in community samples of men who have sex with men. Sex Transm Infect.

[25] Elliot E, McCowan A, McCormak S, Rossi M, Van Every T (2012). O20 Home sampling through social network websites: can we reduce undiagnosed HIV?. Sex Transm Infect.

[26] Fisher MWS, Smith H, Llewellyn C, Alexander S, Ison C (2015). Home sampling for sexually transmitted infections and HIV in men who have sex with men: a prospective observational study. PLoS One.

[27] Saunders J, Mercer C, Sutcliffe L, Hart GJ, Cassell J, Estcourt C (2012). Where do young men want to access STI screening? A stratified random probability sample survey of young men in Great Britain. Sex Transm Infect.

[28] Seguin M, Dodds C, Mugweni E, McDaid L, Flowers P, Wayal S, Zomer E, Weatherburn P, Fakoya I, Hartney T et al. Feasibility and Acceptability of self sampling kits to increase the uptake of HIV testing among black Africans in the United Kingdom: The HAUS Study. Health Technology Assessments. 10.3310/hta22220 PMC5949575 29717978

[29] Brady M, Ward P, Ogunyemi I (2013). Home HIV sampling linked to national HIV testing campaigns: a novel approach to improve HIV diagnosis.

[30] Elliot ERM, McCormack S, McOwan A (2016). Identifying undiagnosed HIV in men who have sex with men (MSM) by offering HIV home sampling via online gay social media: a service evaluation. Sex Transm Infect.

[31] Layder D (1998). Sociological practice.

[32] Rabiee F (2004). Focus-group interview and data analysis. Proc Nutr Soc.

[33] Smith M (2016). Africans in Scotland: heterogeneity and sensistivities to HIV.

[34] Dodds C, Sesay M, Gillgower W (2008). The knowledge, the will and the power - a plan of action to meet the HIV prevention needs of Africans living in England.

[35] Michie S, van Stralen M, West R (2011). The behaviour change wheel: a new method for characterising and designing behaviour change interventions. Implement Sci.

[36] Tobi P, Phillips F, Mead C, Lwembe S, Jones R, Ojwang T, Nyabwa J (2013). P4.057 Investigating the HIV knowledge-personal risk awareness gap among black Africans in London, UK. Sex Transm Infect.

[37] Ritchie J, Spencer L, A Bryman (2002). Qualitative data analysis for applied policy research. Analyzing qualitative data.

[38] Palfreeman A, Ong E (2008). UK National guidelines for HIV testing.

[39] Derald Wing S, Capodilupo C, Torino G, Bucceri J, Holder A, Nadal K (2007). Racial microaggressions in everyday life: implications for clinical practice. Am Psychol.

[40] Michie S, Richardson M, Johnston M, Abraham C, Francis J, Hardeman M, Eccles M, Cane J, Wood C (2013). The behavior change technique taxonomy (v1) of 93 hierarchically clustered techniques: building an international consensus for the reporting of behavior change interventions. Ann Behav Med.

[41] Fisher J, Fisher W (1992). Changing AIDS-risk Behaviour. Psychol Bull.

[42] Hickson F, Owuor J, Weatherburn P, Reid D, Hammond G, Jessup K (2009). Bassline 2008–09: assessing the sexual HIV prevention needs of African people in England.

