# Correction to: Acceptability of HIV self-sampling kits (TINY vial) among people of black African ethnicity in the UK: a qualitative study

**DOI:** 10.1186/s12889-018-5775-0

**Published:** 2018-07-12

**Authors:** C. Dodds, E. Mugweni, G. Phillips, C. Park, I. Young, I. Fakoya, S. Wayal, L. McDaid, M. Sachikonye, J. Chwaula, P. Flowers, F. Burns

**Affiliations:** 1 London School of Hygiene and Tropical Medicine, London, UK; 2 University of Glasgow, Glasgow, UK; 3 University College London, London, UK; 4 University of Edinburgh, Edinburgh, UK; 5 UK Community Advisory Board for HIV and iBase, London, UK; 6 BHA for Equality, Manchester, UK; 7 Glasgow Caledonian University, Glasgow, UK

## Correction to BMC Public Health (2018) 18; 499 DOI: 10.1186/s12889-018-5256-5

It has been highlighted that in the original article [1] there is a typesetting mistake in the name of I. Fakoya. This was incorrectly captured as F. Fakoya. This correction article clarifies the correct name of the author.
