# Supplementary material for: Acceptability of HIV self-sampling kits (TINY vial) among people of black African ethnicity in the UK: a qualitative study
Source: BMC Public Health. 2018 Apr 13;18:499. doi: 10.1186/s12889-018-5256-5 (PMC5899406; doi:10.1186/s12889-018-5256-5)
Supplement: Supplementary file 1 — Thematic coding hierarchy. (PDF 426 kb) [file 12889_2018_5256_MOESM1_ESM.pdf]

Thematic Coding Hierarchy

TESTING IN GENERAL		
	Barriers	
	Shifts in perceptions of testing	
TRUST		
STIGMA		
PRIVACY		
TARGETING OF HSKs TO BLACK AFRICAN PEOPLE		
	Avoiding targeting	
	Limitations	
	Strengths	
SELF TESTING IN GENERAL		
SELF SAMPLING		
	DRIED BLOOD SPOT KITS	
		Limitations
		Strengths
	TINY VIAL KITS	
		Likely users of SSK
		Limitations
		People who would potentially struggle
		Strengths
INFORMATION AND INSTRUCTIONS		
	Additional information - result management	
	Additional information - benefits of free treatment or living well	
	Additional information- video or DVD and web based resources	
	Additional information-Local support services	
	Additional information-Meaning of Results	
	Additional support - phone line for pre-test anxiety and questions	
	Additional support - recorded voice instructions	
	Additional support - trainers need to show how SSK is used	
	Font size	
	Language	
	Number of instructions	
	Pretest counselling	
DISTRIBUTION		
	Barbershops or Hairdressers	
		Limitations
		Role in signposting
		Strengths
	Community Based organisations - specialist organisation	
		Additional resources required (staff

		training, cost, time)
		Kit collection or distribution
		Limitations
		Procedures
		Role in signposting
		Sample Return
		Strengths
	Community Centres- not specialist org or individual	
		Limitations
		Role in signposting
		Strengths
	Dental Surgeries	
		Limitations
		Strengths
	Faith Based Settings	
		Limitations
		Role in signposting
		Strengths
	GP Practice	
		Additional resources required (staff training, cost, time)
		Kit collection or kit distribution
		Limitations
		Procedures
		Sample Return
		Strengths
	HIV Prevention Outreach Workers	
		Additional resources required (staff training, cost, time)
		Kit collection or distribution
		Limitations
		Procedures
		Role in signposting
		Sample Return
		Strengths
	Home	
		Limitations
		Strengths
	Other settings	
		distribution at sexual health centres
		Distribution in local businesses or markets or nightclubs
		Role in signposting
	Personal interaction at settings	
	Pharmacy	
		Additional resources required (staff training, cost, time)
		Kit collection or distribution
		Limitations
		Procedures
		Sample Return
		Strengths
	Universities	

		Limitations
		Role in signposting
		Strengths
	Ranking of settings	
LOCATIONS FOR SAMPLE RETURN		
	Any SSK distributor	
	Individuals post sample to lab	
	Other signposting organisations or businesses	
LOCATIONS FOR TAKING THE SSK SAMPLE		
	Consultation Room	
	Home	
	Office	
	Toilet	
PACKAGING		
CLINICAL GOVERNANCE		
	Additional Testing	
	HIV prevention or risk reduction advice	
	Involvement of health professionals	
	Patient Management	
	Personal Information at KIT collection	
	Provision for those whose first language is not English	
	Results Management	
CONTEXTUAL ISSUES		
	Domestic Violence	
	Health promotion	
	Other	
